# Editorial: Rising stars in space microbiology: 2022

**DOI:** 10.3389/fmicb.2023.1322924

**Published:** 2023-11-13

**Authors:** André Antunes, D'Arcy R. Meyer-Dombard

**Affiliations:** ^1^State Key Laboratory of Lunar and Planetary Sciences, Macau University of Science and Technology, Taipa, Macao SAR, China; ^2^China National Space Administration (CNSA), Macau Center for Space Exploration and Science, Taipa, Macao SAR, China; ^3^Blue Marble Space Institute of Science, Seattle, WA, United States; ^4^Department of Earth and Environmental Sciences, University of Illinois Chicago, Chicago, IL, United States

**Keywords:** space microbiology, Astrobiology, microbial monitoring, pre-biotic life, space exposure experiments of microbes, Astromycology

## Introduction

Human presence in space is inextricably linked with microbes. The topic of “Space Microbiology” emerged the moment we stepped into space (Antipov et al., 1962; Hotchin et al., 1968), as microorganisms are borne in us, on us, and on everything we interact with, and the capacity for microorganisms to survive conditions in the space environment were tested as early as the 1930s (reviewed in Taylor, 1974). Today, the topic has broadened, is extensively linked with aspects of the field of Astrobiology, and is increasingly of paramount importance as humans push our explorations deeper into the Solar System.

The present Research Topic was initiated to highlight work involving young authors, the rising stars in the field of Space Microbiology. It includes five papers, which highlight some of the most dynamic areas within the field, including discussions ranging from the effects of exposure experiments (to spaceflight conditions, and to Mars-like conditions), to the study of pre-biotic life, and to new approaches in microbial monitoring. The Research Topic was well received, with a total of 40 contributing authors from institutions in five different countries spread across the globe (China, Germany, Japan, Italy, and USA), reflecting the wider reach, and interest of this Research Topic.

## Exposure to spaceflight conditions

A growing pool of scientific literature highlights that exposure to spaceflight specific conditions (e.g., microgravity) can lead to increased risks to crews (Simões and Antunes, 2021). These result from a combination of reduced immune response in humans (Sonnenfeld and Shearer, 2002), coupled with several changes in microbial physiology and resistance behavior namely by enhanced biofilm formation, decreased antibiotic susceptibility and increased virulence factors' expression (Mauclaire and Egli, 2010; Tirumalai et al., 2019).

The contribution from Siems et al. focused on this topic by aiming to confirm if spaceflight changes could manifest in human-associated bacteria. For this, they compared the type strain of *Staphylococcus capitis* with three spaceflight relevant isolates (retrieved from the ISS, a clean room, and an artificial gravity bedrest study). Their study looked at changes in growth, colony morphology, metabolism, fatty acid and polar lipid patterns, biofilm formation, susceptibility to antibiotics and survival in different stress conditions, while coupling these with genome-based sequencing and analysis.

Detected phenotypic and genomic differences were mainly observed when compared with the type strain, rather than among the other strains and these were not necessarily indicative of increased virulence. Worth highlighting, the strains showed similar metabolic patterns and the same susceptibility to antibiotics. Nonetheless, some of the phenotypic differences are relevant and require further research within the human spaceflight context. This study confirms that combining classical microbiological methods with genetic analysis allows detailed assessment of the potential threat that specific microbes pose when in spaceflight environments. Such a combined approach will pave the way to further studies and breakthroughs with impacts on future human presence in space.

It should be noted that studying the microbial exposure to space-like conditions is not restricted to the prokaryotic domains. Indeed, the study of fungi in the context of space is also seen as increasingly important in the context of human presence beyond our planet, and as a fundamental piece of the new research field of Astromycology (Simões et al., 2023). Despite their recognized relevance in the context of space exploration, fungi remain understudied.

To help to address this gap, the contribution from Cortesão et al. looked specifically into the issue of fungal growth and surface contamination in spaceflight conditions. They used different strains of *Aspergillus niger* and exposed them to simulated microgravity, comparing it with growth under normal gravity. This species of filamentous fungi was selected as it is known for its relevance for biotechnology and medicine (Sugui et al., 2014; Cairns et al., 2018); it is also commonly reported as colonizer of indoor habitats such as the International Space Station (ISS) (Vesper et al., 2008). The colonizing and biodegrading capabilities of *A. niger*, which extend to a wide range of surfaces, can pose a serious risk to human health and habitat safety both on our planet and beyond. This study uncovered that simulated microgravity led to strain-dependent differences, and affected colony growth, increasing biofilm thickness and spore production. These are essential points, given that surface contamination relies on these two key-features of the fungal colony: spores and biofilm. The results revealed that simulated microgravity did not have an inhibitory effect on the growth of *A. niger* showing instead a potential increase in surface-colonization. As highlighted by the authors, further studies addressing fungal growth and surface contaminations in spaceflight should be prioritized, not only to reduce the negative impact (human health and spacecraft material integrity), but also maximize positive effects from fungal-based biotechnology and in situ resource utilization (ISRU).

## Exposure to planetary-like conditions

When discussing different environmental “extremes” that may be relevant in Astrobiological studies, high salinities are of utmost importance (DasSarma, 2006; Oren, 2014; Antunes et al., 2020; Wu et al., 2022). Contrary to original perceptions, high salt concentrations do not preclude life, as attested by the microbial abundance and diversity in different hypersaline sites ranging from evaporation ponds, to salted food, or even deep-sea brines (Oren, 2002; Antunes, 2017; DasSarma and DasSarma, 2017). Furthermore, salt crystals can even act as potential preservers of microbes and biomolecules, and biomarkers over geological timescales (Gramain et al., 2011; Stan-Lotter and Fendrihan, 2015; Wilhelm et al., 2017).

Regarding the specific settings of Mars, the relatively recent identification of perchlorate salts in regolith and their widespread distribution (Kounaves et al., 2014; Clark and Kounaves, 2016) raises several issues, particularly as these salts have been hypothesized as a critical chemical hazard for putative life forms (Davila et al., 2013; Wadsworth and Cockell, 2017). Perchlorates have been poorly studied, as environments with high concentrations of these salts are scarcely reported on Earth. This is a key issue in the field.

The contribution from Cassaro et al. addresses our limited knowledge on the effects of exposure to perchlorates by studying the polyextremophilic black fungus *Cryomyces antarcticus*, a eukaryotic model organism isolated from cryptoendolithic communities in the Antarctic. The presence of perchlorates in soil and ice has been previously confirmed in the McMurdo Dry Valleys in Antarctica (considered one of the best terrestrial analogs for Mars: Kounaves et al., 2010), making this a well-suited candidate species for testing. In this study, the resistance of *C. antarcticus* was assessed when grown on different hypersaline substrata and when grown on Martian relevant perchlorate medium. The results report a good survivability and metabolic activity recovery of this model organism when exposed to these Mars-relevant conditions. These outcomes have implications on our understanding of Life's resilience when exposed to under-studied Mars-like conditions, with ramifications on the search for Life, on planetary protection, and on potential use of microbes for ISRU on Mars.

## Pre-biotic life

Space Microbiology is also linked to other important research threads including the search for new insights into the origin of life. A vital prerequisite for prebiotic chemistry is the significant accumulation of critical building blocks of life. It is suggested that more frequent impacts on the primordial Earth could have induced a more reducing steam atmosphere (Zahnle et al., 2020). The true effects and constraints of such a setting are not fully clear as they would favor global spreading and efficiency in the synthesis of life building blocks yet also threaten the stability of organics and their significant accumulation (Miyakawa et al., 2002; Pearce et al., 2022).

In their contribution, Zhang et al. looked into this gap. The authors used a thermodynamic tool to analyze the synthesis affinity of various life building blocks using inorganic gasses as reactants at elevated temperatures and corresponding steam pressures relevant with the steam-seawater interface. The results of this study suggest that reducing conditions induced by primitive impacts could provide propitious conditions for the synthesis/accumulation of some of life's building blocks, although this was not necessarily the case for some critical species (e.g., HCN and nucleosides), which were not favored to accumulate to appreciable levels. The insights from this study bring us a step closer to unveiling the processes involved in the origin of life, as well as help to guide future efforts to search for or understand the stability of biomolecules on Mars, the icy moons of the outer solar system, or elsewhere. One important future research direction highlighted by the authors is studying craters formed by more reducing impactors to look for preservation of prebiotic materials.

## New approaches in microbial monitoring

As we enter a new era of space exploration, there is a difficult balance between incorporating new developments, satisfying increased needs regarding analytical measurements and lab-based experiments, and reducing payload requirements. In this context, lab-on-a-chip nanobiosensors appear to be an emerging and high-relevance technology, given their low footprint, high accuracy, and low payload requirements. In the contribution by Cinti et al., the current and potential roles such sensors in space exploration and in extreme environment investigation is reviewed, reporting what has been tested so far, and clarifying the direction toward which these newly developed technologies are heading for regarding exploration in extreme environments and in space. Despite being rarely reported, such sensors will play a key role in the future of Space Microbiology and are expected to significantly improve the quality of long-term space missions.

## Final remarks

Space Microbiology is the subject of intense interest and activity by researchers across different areas of knowledge and spread across the globe. The next few years of research centered on Earth, Moon, Mars, and various icy ocean worlds, are expected to deliver significant new developments on this cross-disciplinary field.

## Author contributions

AA: Conceptualization, Data curation, Funding acquisition, Resources, Supervision, Validation, Writing—original draft, Writing—review & editing. DRMD: Conceptualization, Data curation, Funding acquisition, Resources, Supervision, Validation, Writing—original draft, Writing—review & editing.

## References

[B1] Antipov V. V. Bayevskiy R. M. Gazenko O. G. Genin A. M. Gyurdzhian N. N. Zhukov-Verezhnikov N. N.. (1962). “Some results of medical and biological investigations in the second and third satellites,” in Problems of Space Biology, Vol. 1, ed N. M. Sisakyan (Moscow; Washington, DC: National Aeronautics and Space Administration). Available online at: https://play.google.com/store/books/details?id=4aT9-2uUWNIC&rdid=book-4aT9-2uUWNIC&rdot=1

[B2] Antunes A. (2017). “Extreme Red Sea: life in the deep-sea anoxic brine lakes,” in Human Interaction with the Environment in the Red Sea, eds D. A. Agius, E. Khalil, E. Scerri, and A. Williams (Leiden: Brill), 30–47. 10.1163/9789004330825_004

[B3] Antunes A. Olsson-Francis K. McGenity T. J. (2020). Exploring deep-sea brines as potential terrestrial analogues of oceans in the icy moons of the outer solar system. Curr. Issues Mol. Biol. 38, 123–162. 10.21775/cimb.038.123 31967579

[B4] Cairns T. C. Nai C. Meyer V. (2018). How a fungus shapes biotechnology: 100 years of *Aspergillus niger* research. Fungal Biol. Biotechnol. 5, 13. 10.1186/s40694-018-0054-5 29850025 PMC5966904

[B5] Clark B. C. Kounaves S. P. (2016). Evidence for the distribution of perchlorates on Mars. Int. J. Astrobiol. 15, 311–318. 10.1017/S1473550415000385

[B6] DasSarma S. (2006). Extreme halophiles are models for astrobiology. Microbe Mag. 1, 120–126. 10.1128/microbe.1.120.1

[B7] DasSarma S. DasSarma P. (2017). “Halophiles,” in eLS (Hoboken, NJ: John Wiley and Sons, Ltd), 1–13.

[B8] Davila A. F. Willson D. Coates J. D. McKay C. P. (2013). Perchlorate on mars: a chemical hazard and a resource for humans. Int. J. Astrobiol. 12, 321–325. 10.1017/S1473550413000189

[B9] Gramain A. Díaz G. C. Demergasso C. Lowenstein T. K. McGenity T. J. (2011). Archaeal diversity along a subterranean salt core from the Salar Grande (Chile). Environ. Microbiol. 13, 2105–2121. 10.1111/j.1462-2920.2011.02435.x 21355972

[B10] Hotchin J. Lorenz P. Hemenway C. L. (1968). The survival of terrestrial microorganisms in space at orbital altitudes during Gemini satellite experiments. Life Sci. Space Res. 6, 108–114. 11982025

[B11] Kounaves S. P. Chaniotakis N. A. Chevrier V. F. Carrier B. L. Folds K. E. Hansen V. M.. (2014). Identification of the perchlorate parent salts at the Phoenix Mars landing site and possible implications. Icarus 232, 226–231. 10.1016/j.icarus.2014.01.016

[B12] Kounaves S. P. Stroble S. T. Anderson R. M. Moore Q. Catling D. C. Douglas S.. (2010). Discovery of natural perchlorate in the Antarctic dry valleys and its global implications. Environ. Sci. Technol. 44, 2360–2364. 10.1021/es9033606 20155929

[B13] Mauclaire L. Egli M. (2010). Effect of simulated microgravity on growth and production of exopolymeric substances of *Micrococcus luteus* space and earth isolates. FEMS Immunol. Med. Microbiol. 59, 350–356. 10.1111/j.1574-695X.2010.00683.x 20482631

[B14] Miyakawa S. James Cleaves H. Miller S. L. (2002). The cold origin of life: a implications based on the hydrolytic stabilities of hydrogen cyanide and formamide. Orig. Life Evol. Biosph. 32, 195–208. 10.1023/A:1016514305984 12227424

[B15] Oren A. (2002). Diversity of halophilic microorganisms: environments, phylogeny, physiology, and applications. J. Ind. Microbiol. Biotechnol. 28, 56–63. 10.1038/sj/jim/7000176 11938472

[B16] Oren A. (2014). Halophilic archaea on Earth and in space: growth and survival under extreme conditions. Philos. Trans. R. Soc. A Math. Phys. Eng. Sci. 372, 20140194. 10.1098/rsta.2014.0194 25368347

[B17] Pearce B. K. D. Molaverdikhani K. Pudritz R. E. Henning T. Cerrillo K. E. (2022). Toward RNA life on early Earth: from atmospheric HCN to biomolecule production in warm little ponds. Astrophys. J. 932, 9. 10.3847/1538-4357/ac47a1

[B18] Simões M. F. Antunes A. (2021). Microbial pathogenicity in space. Pathogens 10, 450. 10.3390/pathogens10040450 33918768 PMC8069885

[B19] Simões M. F. Cortesão M. Azua-Bustos A. Bai F.-Y. Canini F.. (2023). The relevance of fungi in astrobiology research – Astromycology. Mycosphere 14, 1190–1253. 10.5943/mycosphere/14/1/13

[B20] Sonnenfeld G. Shearer W. T. (2002). Immune function during space flight. Nutrition 18, 899–903. 10.1016/S0899-9007(02)00903-6 12361785

[B21] Stan-Lotter H. Fendrihan S. (2015). Halophilic *Archaea*: life with desiccation, radiation and oligotrophy over geological times. Life 5, 1487–1496. 10.3390/life5031487 26226005 PMC4598649

[B22] Sugui J. A. Kwon-Chung K. J. Juvvadi P. R. Latgé J.-P. Steinbach W. J. (2014). *Aspergillus fumigatus* and related species. Cold Spring Harb. Perspect. Med. 5, a019786. 10.1101/cshperspect.a019786 25377144 PMC4315914

[B23] Taylor G. R. (1974). Space microbiology. Annu. Rev. Microbiol. 28, 121–137. 10.1146/annurev.mi.28.100174.001005 4215364

[B24] Tirumalai M. R. Karouia F. Tran Q. Stepanov V. G. Bruce R. J. Ott C. M.. (2019). Evaluation of acquired antibiotic resistance in *Escherichia coli* exposed to long-term low-shear modeled microgravity and background antibiotic exposure. mBio 10, e02637-18. 10.1128/mBio.02637-18 30647159 PMC6336426

[B25] Vesper S. J. Wong W. Kuo C. M. Pierson D. L. (2008). Mold species in dust from the International Space Station identified and quantified by mold-specific quantitative PCR. Res. Microbiol. 159, 432–435. 10.1016/j.resmic.2008.06.001 18602989

[B26] Wadsworth J. Cockell C. S. (2017). Perchlorates on Mars enhance the bacteriocidal effects of UV light. Sci. Rep. 7, 4662. 10.1038/s41598-017-04910-3 28684729 PMC5500590

[B27] Wilhelm M. B. Davila A. F. Eigenbrode J. L. Parenteau M. N. Jahnke L. L. Liu X. L.. (2017). Xeropreservation of functionalized lipid biomarkers in hyperarid soils in the Atacama Desert. Org. Geochem. 103, 97–104. 10.1016/j.orggeochem.2016.10.015 29743757 PMC5937136

[B28] Wu J.-H. McGenity T. J. Rettberg P. Simões M. F. Li W.-J. Antunes A. (2022). The archaeal class *Halobacteria* and astrobiology: knowledge gaps and research opportunities. Front. Microbiol. 13, 1023625. 10.3389/fmicb.2022.1023625 36312929 PMC9608585

[B29] Zahnle K. J. Lupu R. Catling D. C. Wogan N. (2020). Creation and evolution of impact-generated reduced atmospheres of early Earth. Planet. Sci. J. 1, 11. 10.3847/PSJ/ab7e2c

